# Gender stereotypes across the ages: On-line processing in school-age children, young and older adults

**DOI:** 10.3389/fpsyg.2015.01388

**Published:** 2015-09-22

**Authors:** Anna Siyanova-Chanturia, Paul Warren, Francesca Pesciarelli, Cristina Cacciari

**Affiliations:** ^1^School of Linguistics and Applied Language Studies, Victoria University of WellingtonWellington, New Zealand; ^2^Department of Biomedical, Metabolic and Neurological Sciences, University of Modena and Reggio EmiliaModena, Italy

**Keywords:** gender stereotypes, on-line language processing, implicit measure, children, young adults, older adults

## Abstract

Most research to date on implicit gender stereotyping has been conducted with one age group – young adults. The mechanisms that underlie the on-line processing of stereotypical information in other age groups have received very little attention. This is the first study to investigate real time processing of gender stereotypes at different age levels. We investigated the activation of gender stereotypes in Italian in four groups of participants: third- and fifth-graders, young and older adults. Participants heard a noun that was stereotypically associated with masculine (*preside* “headmaster”) or feminine roles (*badante* “social care worker”), followed by a male (*padre* “father”) or female kinship term (*madre* “mother”). The task was to decide if the two words – the role noun and the kinship term – could describe the same person. Across all age groups, participants were significantly faster to respond, and significantly more likely to press ‘yes,’ when the gender of the target was congruent with the stereotypical gender use of the preceding prime. These findings suggest that information about the stereotypical gender associated with a role noun is incorporated into the mental representation of this word and is activated as soon as the word is heard. In addition, our results show differences between male and female participants of the various age groups, and between male- and female-oriented stereotypes, pointing to important gender asymmetries.

## Introduction

Gender stereotyping, for better or worse, occurs frequently in everyday life. We seem to readily attribute masculine gender to doctors, surgeons, and politicians, and feminine gender to nurses, school teachers, and secretaries. When our personally held beliefs are compromised in one way or another, we feel obliged to provide additional information, as suggested by terms like *male nurse* or *female soldier*. This occurs even when other clues already point to the gender of the referent, as in the following example: *Military rules ban pregnant servicewomen from front-line duties, though last year another female British soldier gave birth two weeks after returning from her six-months deployment to Afghanistan* [BBC News, 24th March, 2013, emphasis added].

In contexts where there is no explicit information about the gender associated with a specific occupation (*doctor, nurse*), personal trait (*aggressive, nurturing*), or activity (*mending, laundering*), we rely on our beliefs and background knowledge to infer – sometimes erroneously – the more likely gender. A wealth of psycholinguistic studies has looked at the activation of stereotypical gender information during language processing. Specifically, it has been widely documented that when language users encounter stereotypically incongruent information (male nurse or female doctor), their processing slows down (Banaji and Hardin, 1996; Carreiras et al., 1996; Garnham et al., 2002; Duffy and Keir, 2004; Oakhill et al., 2005; Cacciari and Padovani, 2007; Kreiner et al., 2008; Pyykkönen et al., 2010; Siyanova-Chanturia et al., 2012). These studies have shown that stereotypical gender information is incorporated into the mental representation of the role noun in question (*doctors/surgeons/politicians* are assumed to be males, while *nurses/teachers/secretaries* are assumed to be females), and that gender activation occurs at the time a role noun is encoded (Oakhill et al., 2005; Siyanova-Chanturia et al., 2012). These and other studies have used a range of methodologies, paradigms, and tasks to investigate moment-by-moment processing of stereotypical gender information, predominantly in young adults. As we will see in the following review of previous research, the processing of stereotypical gender violations in other age groups – such as children and older adults – remains poorly understood. There is also relatively little data that indicate whether the stereotypicality effects vary with the sex of the participants or with the gender indicated by the linguistic items involved.

### Young Adults

The bulk of the research on the processing of gender stereotypes has focused on young adults, and has shown that linguistic information congruent with stereotypes is processed more rapidly than incongruent information. An early study (Banaji and Hardin, 1996) measured response times in judging the grammatical gender of personal pronouns (*he, she*) that followed prime words that were gender-biased either definitionally (*mother, father*) or because of stereotyped use (*nurse, doctor*). Responses were slower when there was a mismatch between the gender of the prime and that of the pronoun, especially for definitional terms (*mother* followed by *he*). Participants also responded significantly more quickly to targets that matched their own gender. Banaji and Hardin’s (1996) paradigm has since been used in other behavioral studies and in studies measuring event-related brain potentials (ERPs; Cacciari and Padovani, 2007; Siyanova-Chanturia et al., 2012). Interestingly, Cacciari and Padovani (2007) found a stereotype incongruency effect with masculine pronouns (*secretary-he*) but not with feminine pronouns (*engineer-she*).

In a study employing a similar paradigm, and one on which the current study is based, Oakhill et al. (2005) asked participants to read word pairs in which a stereotypically male or female role noun (*engineer* or *secretary*, respectively) was followed by a kinship term that was either congruent (*engineer* – *brother*) or incongruent (*engineer* – *sister*), and to decide for each pair whether they could be used to refer to the same person. Participants responded more rapidly to congruent than incongruent word pairs, even when they were explicitly instructed to suppress their gender stereotypes.

In an eye-tracking study (Duffy and Keir, 2004), test sentences contained masculine and feminine role nouns as antecedents to stereotypically congruent or incongruent reflexive pronouns (*The electrician taught himself/herself …*). Test sentences were preceded either by a discourse context specifying the sex of the referent or by a sex-neutral context. In the neutral contexts, automatic activation of gender stereotypical information encoded in the role nouns resulted in higher processing costs and longer fixation times when the test sentences contained incongruent pronouns (for similar results, see Irmen, 2007; Pyykkönen et al., 2010; also see Esaulova et al., 2014). However, when the preceding context signaled that the character’s sex matched the reflexive pronoun, the incongruency effect disappeared. Kreiner et al. (2008) similarly found congruency effects on fixation times in anaphoric sentences (where the reflexive follows the noun to which it refers: *Yesterday the minister left London after reminding himself/herself about the letter*, where the reflexive pronoun refers to a preceding noun) but not in cataphoric sentences (where the reflexive precedes its noun: *After reminding himself/herself, the minister immediately went to the meeting at the office*).

ERP studies have shown that the brain response to gender stereotype violations in language might be indexed by two different components, the N400 and the P600. The N400 is a negative-going deflection peaking around 400 ms. after stimulus onset that has traditionally been shown to reflect semantic and world knowledge violations (for an overview, see Kutas and Federmeier, 2011). The P600 is a slow positive shift emerging 500–900 ms. after stimulus onset, traditionally associated with syntactic violations, but also linked to semantic anomaly (Osterhout and Holcomb, 1992, 1995; Kuperberg et al., 2003; Kim and Osterhout, 2005; Bornkessel-Schlesewsky and Schlesewsky, 2008). White et al. (2009) presented participants with a gender category (*men/women*) followed by a word stereotypically associated with males (*aggressive*) or females (*nurturing*). Participants judged whether or not the two words matched, according to their beliefs about gender stereotypes. Stereotypically incongruent combinations (*men/nurturing, women/aggressive*) elicited a larger N400 than congruent ones. In their ERP study, Siyanova-Chanturia et al. (2012) used Banaji and Hardin’s (1996) paradigm described above with native speakers of Italian. Participants judged the grammatical gender of a personal pronoun (equivalent to English *he, she*) following either a definitionally gendered noun (*mother, father*) or a gender stereotyped role noun (*teacher, driver*). After definitionally gendered nouns, incongruent pronouns (*mother/he* or *father/she*) resulted in a N400 effect, but after stereotypically gendered nouns, this effect was found only with male targets (*teacher/he* but not *driver/she*), suggesting that participants were more accepting of female drivers than male teachers.

In an earlier ERP study of gender stereotypes, Osterhout et al. (1997) observed larger P600s when the stereotypical gender of an antecedent role noun was incongruent with the gender of a reflexive pronoun (*doctor – herself*) than when it was congruent. They found a stronger effect for female than for male participants, suggesting that females have stronger gender stereotypes. Finally, Irmen et al. (2010) conducted a study in German, in which participants read statements involving occupations (*florists, pilots*) followed by masculine, feminine, or neutral anaphoric noun phrases (*these men/women/people*). While the occupations are stereotypically associated with males or females, all of the nouns representing them had masculine grammatical gender. When the anaphors were semantically incongruent with their antecedents, feminine anaphors produced more positive P600 responses than masculine anaphors. Irmen et al. (2010) suggested this was because the masculine anaphors were congruent at least with the masculine grammatical gender of the female antecedent noun, and that this eased integration, compared with the feminine anaphors.

A range of behavioral, eye-tracking, and ERP studies have thus shown stereotypical gender effects in experiments with young adults. These studies suggested that information about stereotypical gender – denoting an occupation or a personal characteristic – is incorporated into the reader’s representation of a word, and that this information is difficult to suppress during on-line language processing. A few of these studies have also reported asymmetries that depend on the gender indicated by the words involved and on the sex of the participant, with stronger incongruency effects reported for combinations of female stereotypes with masculine pronouns and from female participants.

### Older Adults

Most of the stereotype research with older populations has focused on racial rather than gender stereotypes, with older adults frequently found to be more prejudiced than younger adults. Following Devine (1989), it has been widely hypothesized firstly that what sets apart prejudiced and non-prejudiced individuals is the extent to which they are able to suppress stereotyped behavior, and secondly that this ability diminishes with age. For instance, von Hippel et al. (2000) found that younger (18–25 years of age) but not older (65–95) adults were able to ignore racial stereotypes when rating the intelligence of two characters presented as African American and Caucasian. Similarly, Gonsalkorale et al. (2009) found that older adults showed greater implicit bias because of their inability – relative to young adults – “to regulate automatically activated associations” (p. 412), and Radvansky et al. (2010) found that older adults (60–88) drew on and maintained racial stereotypic references to a much greater extent than younger adults (18–25).

To the best of our knowledge, only one study has investigated the processing of gender stereotypical information in older adults. In a self-paced reading study (Radvansky et al., 2009), younger (18–22) and older (60–87) adults read a series of short stories (adapted from Duffy and Keir, 2004). Critical sentences contained sequences such as *The babysitter/plumber found herself/himself …* in which the reflexive pronoun was either congruent or incongruent with the gender stereotyped occupation of the character. Both young and older adults showed an effect of congruency on reading time. But unlike the findings in studies on racial prejudice, both groups of adults were found to be capable of suppressing gender stereotypes when counter-stereotypic information was provided in the preceding context. There is no indication as to whether this varied with participant sex or with the gender of the stereotyped items.

So while research on racial stereotypes suggests that older adults may be less able to suppress the activation of stereotypical information than younger adults, the small amount of relevant research suggests that this may not be the case with gender stereotypes.

### Children

Gender stereotyping in children has received increasing attention in recent years, with a particular focus on the development of stereotype behavior during childhood. Hill and Flom (2007) found sensitivity to gender stereotypes at 24 months but not at 18 months, using a preferential looking paradigm in which children watched male and female actors performing masculine and feminine stereotypical activities. An earlier study (Poulin-Dubois et al., 2002) used a generalized imitation paradigm in which children selected a male and a female doll to imitate masculine and feminine stereotypical activities. They found that 24-month-old girls, but not boys, were sensitive to the violation of gender stereotypical activities.

Research with children has also addressed the question of *stereotypical gender asymmetry*, that is, whether gender stereotyping is less restrictive for female than for male stereotypes, as predicted, for instance, by Social Role theory (Eagly and Steffen, 1984; Diekman and Eagly, 2000; Eagly et al., 2000). Wilbourn and Kee (2010) asked 8- and 9-year-old children to create sentences that paired male and female proper names with stereotypically masculine and feminine occupations. The results showed that children were less likely to think of males engaging in traditional feminine activities (*Henry-nurse*) than the other way around (*Mary-doctor*). As noted above, a similar asymmetry has recently been found in young adults (Siyanova-Chanturia et al., 2012, see also Cacciari and Padovani, 2007; Irmen et al., 2010; Reali et al., 2014).

Banse et al. (2010) considered both stereotype knowledge and stereotype flexibility in groups of 5-, 8-, and 11-year-old children. Stereotype knowledge is reflected in automatic stereotyping that occurs independently of whether the individual considers the stereotypes to be accurate or not, while stereotype flexibility involves a recognition that stereotypes can be wrong (see also Signorella et al., 1993; Trautner et al., 2005). The children were assessed on how they associated gender-stereotyped common objects (*iron, hammer*) with men and women, and gender-stereotyped toys (*doll, truck*) with boys and girls. The results showed that gender stereotype knowledge for toys was at ceiling as early as 5 years of age, and for common objects reached ceiling levels by 11 (while already very high at five). Stereotype flexibility, that is, the realization that stereotypes are not immutable, showed a considerable increase from the age of 5–11, and, unlike stereotype knowledge, was higher at all ages for common objects than for toys. Differences between girls and boys and between female- and male-related stereotypes were reported neither for stereotype knowledge nor for stereotype flexibility.

Few studies in this area tap into the processes underlying the moment-by-moment comprehension of gender stereotypes. A notable exception is Most et al. (2007), who used an auditory Stroop paradigm in which young adults and third-graders (∼9 years old) categorized the sex of voices that pronounced male and female proper names, or stereotypically male (*football*), female (*makeup*), or neutral (*paper*) words. Both children and adults were slower when the voice’s sex was incongruent either with the gendered stereotype of the spoken word (*makeup* spoken with a male voice; *football* spoken with a female voice) or with the gender of the proper name (*Cindy* spoken with a male voice; *Jason* spoken with a female voice). This suggests that implicit gender associations are already present in 8- to 9-year-old children. Unfortunately, it is not clear how the gender stereotypicality of the heterogeneous types of target words (nouns, adjectives, verbs, names of activities, objects, professions, concrete, and abstract words) was established. Nor does there seem to be any control of the lexical properties that are known to affect the time it takes to decode a word stimulus (such as word frequency, length, etc.).

The studies cited above provide a sketch of the development of gender stereotypical behavior in children. Automatic stereotyping is evident from an early age and firmly in place by about 11 years. At the same time, children show evidence from 5 to 11 that they are increasingly able to override their stereotype behaviors. Stronger sensitivity to stereotype violations has been reported for very young girls than for boys, and there is some evidence that children are more sensitive to gender incongruencies in which stereotypically feminine roles are paired with male persons.

## The Present Study

The majority of the studies on gender stereotyping conducted with children and older adults employed explicit off-line measures such as questionnaires, off-line reading, and judgment and classification tasks. Although such measures usefully elucidate social beliefs and attitudes, they do not provide information on the underlying moment-by-moment processes that can be revealed by real-time measures such as reaction times, eye-tracking, and ERPs – methodologies that have so far been mostly used with young adults. In addition, previous studies have had little to say either on the possible differences between the sexes in terms of behavior with gender stereotypes, or on the possibility that female- and male-gendered language may be responded to differently.

The present study therefore aimed to use the same real-time measure to assess gender stereotype behavior with a range of ages, namely third- and fifth-graders (∼8 and 10 years of age), young adults (mean age of 24), and older adults (mean age of 77). It also aimed to assess differences between female and male participants and between female- and male-gendered stereotypes. To achieve this, we adapted Oakhill et al.’s (2005) paradigm outlined earlier. Participants had to decide whether two words – a gender-biased occupational role and a kinship term – could describe the same person. The two terms formed either a stereotypically congruent pair (*engineer* – *brother*) or an incongruent pair (*secretary – father*). Our adaptation of the paradigm was that we used auditory rather than visual presentation of the stimuli, since this seemed better suited for testing participants with different reading abilities. Response choices (‘yes’/‘no’) and decision times for those choices were collected.

Our predictions are that all age groups will show sensitivity to the violation of gender stereotypical information, but that the extent of this sensitivity will be age-dependent. In particular, we predict that adults will show greater stereotype flexibility and be better able than children to suppress gender stereotypes and therefore to accept the incongruent role-kinship pairs as possibly referring to the same person. The evidence from racial stereotypes indicates that older adults are less well able to suppress stereotypes than younger adults, while Radvansky et al. (2009) suggest that this may not be the case for gender stereotypes. It remains an empirical question, therefore, whether the results for older adults will show the same or lowered rates of suppression of gender stereotypes compared with those for young adults. We will look to the extent and speed of acceptance that the incongruent pairs may refer to the same person as a measure of this. Within the two groups of children, we predict that the change in stereotype flexibility demonstrated by Banse et al. (2010) for children between the ages of 5 and 11 will be reflected in stronger and more rapid acceptance of incongruent pairs by our fifth-graders than by our third-graders.

As far as the gender of the tested words is concerned, we note that the research reviewed above showed asymmetries both for 8- and 9-year-old children (Wilbourn and Kee, 2010) and for young adults (Siyanova-Chanturia et al., 2012). We predict that for our data, these groups will be more likely to accept the combination of male roles with female kinship terms (*engineer-sister*) than vice versa (*secretary-brother*). We have no reason not to expect the same of our older adults.

With regard to sex differences between our participants, we predict – on the basis of the study with very young children by Poulin-Dubois et al. (2002) – that young girls will show greater stereotype flexibility than young boys, and will therefore be more likely to accept incongruent pairs. There is little direct evidence cited above that addresses this issue in adults, but our prediction is that by adulthood, male participants will show similar degrees of stereotype flexibility as females.

## Method

### Participants

Our young adult group comprised 28 students at the University of Modena and Reggio Emilia (13 females, mean age: 24.1, range: 20–30, *SD:* 4.3) who participated in the experiment for course credit or a small gift (equivalent of €10).

Our group of older adults was made up of 30 cognitively preserved older adults (14 females, mean age: 77.4, range: 72–82, *SD:* 2.5) with homogenous educational and socio-economic backgrounds. They all achieved a Mini-Mental State Evaluation score (MMSE, Folstein et al., 1975) equal to, or higher than 26 (*M* = 28.2, *SD:* 1.7, range: 26–30) and had at least 10 years of formal education. They did not receive a gift for their participation.

Our two groups of children consisted of 43 third-graders (20 females, mean age: 8.5, range: 7.9–9.5, *SD:* 0.4) and 42 fifth-graders (17 females, mean age: 10.4, range: 9.7–11.2, *SD:* 0.3) from the same school in the province of Modena, Emilia Romagna (Italy). They received a small gift (equivalent of €3) for their participation. The use of these two age groups was based on our review of earlier studies which suggests that these groups fall in a period of development where stereotype flexibility is increasing rapidly (Signorella et al., 1993; Trautner et al., 2005; Banse et al., 2010). We decided not to test children younger than third grade because of the task demands of a paradigm that requires high accuracy and speed.

All participants were residents in the province of Modena, Emilia Romagna (Italy). They were informed of their rights and gave written informed consent for participation in the study (for children, this consent was granted by their parents), according to the Declaration of Helsinki, and in line with the ethical requirements of the University of Modena and Reggio Emilia.

### Materials

Material selection followed two stages of norming, with adults and with children. In all cases rating scales were used, with the scale poles reversed for half of the participants. None of the participants used in the norming studies also took part in the main experiment. An initial set of 260 Italian words (nouns, past participles, and adjectives), morphologically unmarked for gender and specifying occupations, roles and individual characteristics, was presented in two questionnaires (each containing 130 words) to 40 students (20 females). Participants rated the extent to which each word was associated with men, women, or both, using a seven-point scale. From this initial set, 60 words were selected that were rated as highly male-oriented (30 words) or female-oriented (30 words). A further set of 40 participants subsequently rated the valence (positive, negative, or neutral connotations) of the 60 selected words.

To ensure the 60 selected words were familiar to third- and fifth-graders and had gender associations from the children’s perspective, they were included in additional questionnaires presented to 133 children (half third-graders and half fifth-graders; half females). Participants selected from three options, indicating that the words could be used: (1) only for men, (2) for both men and women, (3) only for women. There was a fourth option – ‘I don’t know’ – in case the word was not known to the participant; this option always appeared last. The questionnaire also included filler items morphologically marked for gender (*amico* “male friend”). If a child performed poorly on such items, then their data were excluded from the norming procedure.

On the basis of this norming, we selected nine words that received the highest ratings of male-oriented stereotypicality (*preside* “headmaster”), and nine words that received similarly high ratings of female-oriented stereotypicality (*badante* “social care worker”) in the adult rating task. All 18 selected words were known to both third- and fifth-graders. The male- and female-oriented words did not significantly differ in stereotypicality for either adults or children, nor in their valence. The words in the two groups were also comparable in terms of frequency (*Repubblica* corpus, Baroni et al., 2004), length (number of characters), and in the durations of the recorded tokens used in the experiment (see below). Norming and lexical statistics are summarized in **Table 1**.

**Table 1:** Mean log frequency, length, stereotypicality, valence, and millisecond duration of target stimuli.

	Male stereotype	Female stereotype	*p*
Log frequency	3.1 (2.4–3.7) *0.4*	2.3 (0.0–3.7) *1.2*	=0.11
Length (characters)	8.8 (4.0–13.0) *2.8*	9.3 (7.0–12.0) *1.5*	=0.60
Adult stereotypicality	2.6 (2.0–3.5) *0.5*	2.9 (1.3–3.8) *0.8*	=0.17
Child stereotypicality	1.7 (1.6–1.8) *0.1*	1.8 (1.4–1.9) *0.2*	=0.09
Valence	4.5 (3.8–5.8) *0.6*	4.9 (4.1–5.5) *0.5*	=0.20
Duration (ms)	779 (526–1032) *175.3*	826 (659–972) *107*	=0.22

Range is indicated in parentheses and standard deviation in italics below.

The selected items (see Appendix) used one of three nominal endings not associated with a specific grammatical gender. Each of the groups of male- and female-oriented words contained five words ending in -*ista*, three in -*e*, and one in a consonant.

Following Oakhill et al. (2005), each of the 18 role nouns was paired with each of six paired kinship terms: *sorella* “sister,” *fratello* “brother,” *madre* “mother,” *padre* “father,” *moglie* “wife,” *marito* “husband,” resulting in three stereotypically congruent and three stereotypically incongruent word pairs for each role noun. The words in each kinship pair were comparable in terms of their lexical characteristics (see **Table 2**).

**Table 2:** Mean log frequency, length, and millisecond duration of the six kinship terms used in the experiment.

	*madre padre*	*sorella fratello*	*moglie marito*
Log frequency	4.6 4.8	4.0 4.3	4.4 4.7
Length (characters)	5 5	7 8	6 6
Duration (ms)	440 435	668 680	616 587

The tokens of all words used in the experiment were created using ALFa Reader 3 voice synthesizer software. We used speech production software rather than a human voice to make the recording as neutral as possible (free of regional accents, personal traits, etc.). Two native speakers of Italian judged the recordings to be natural and to have native-like prosody.

### Procedure

Participants were seated comfortably in a silent room. In each trial, a fixation point (+) appeared in the center of a computer screen for 1500 ms. followed by a blank screen for 350 ms. Participants then heard the prime (role noun) and 250 ms. later the target (kinship term), and decided whether the two words could describe the same person. They were instructed to listen carefully to both words and to press the ‘yes’ or ‘no’ button on a button box as quickly and accurately as possible (button positions were reversed for half of the participants). The subsequent trial began after the response. To ensure that the gender of the voice did not bias participants’ response (for such an effect, see Most et al., 2007; Van Berkum et al., 2008), half of the participants listened to the words pronounced with a male-synthesized voice and half with a female-synthesized voice.

The experiment comprised six blocks of 36 trials (216 word pairs in total). Trials were pseudorandomised in each block, but each block contained equal numbers of stereotypically congruent and incongruent pairs, and of feminine and masculine role nouns. Each role noun occurred only once in each block. In addition to the 18 test pairs, each block contained 18 filler pairs (half congruent, half incongruent), whose primes were role nouns morphologically marked for gender (*amico* “male friend,” *ragazza* “girl”). These filler pairs provided a measure of performance accuracy in the task (see below).

The experimental session was preceded by a practice block of 20 trials (half congruent and half incongruent word pairs of the same type as the fillers). After each block, participants were invited to take a short break.

## Analysis and Results

A total of 16 participants (11% of the original 143) were excluded for one or more of the following reasons: they exceeded the 25% error rate threshold on the fillers (*N* = 3), they were non-native speakers of Italian (*N* = 4), they did not follow the instructions (*N* = 3), they were identified as having hearing problems or learning difficulties (*N* = 3) or as not being naïve to the nature of the experiment (*N* = 1), or because of equipment failure (*N* = 2). One further participant was excluded on the basis of having exceptionally long response times (a mean response time more than 2.5 standard deviation from the mean for their age group). Hence the analyses were conducted on 34 third-graders (17 females), 39 fifth-graders (17 females), 26 young adults (13 females), and 27 older adults (14 females). The mean error rate on the fillers for the retained participants was 9.7% for third-graders, 7.8% for fifth-graders, 3.9% for young adults, and 7.9% for older adults. The distribution of response times across both experimental and filler items was examined within each age group, and cut-off times determined for the group. A total of 2.06% of responses were removed.

Mixed effects models were computed over responses to the experimental items for response choice (logistic regression) and response times (linear regression), using the *lme4* package in R (Bates et al., 2015). The *afex* package (Singmann et al., 2015) was used to determine Chi-square values and significance levels for relevant factors. For the response choice analysis, the dependent variable was the selection of ‘yes’ or ‘no’ (prime and target could or could not describe the same person). In the analysis of response times, since these did not follow a normal distribution, the effect of a range of transformations was tested, and the inverse square root function [transformed RT = 1/sqrt(RT)] selected as the best fit to a normal distribution.

Following model comparison, the random effects structure for both response choice and response time analyses included random intercepts for participant, prime (the stereotyped role word) and target (the kinship term), and random slopes by participants across the sequence of blocks in the experiment. The fixed effects were Participant Sex, Age Group (third grade, fifth grade, young adult, or older adult), Block^1^, Target Gender (female or male kinship term), and Congruence (the target word formed a congruent or incongruent pair with the prime word).

### Response Choice Analysis

**FIGURE 1:**
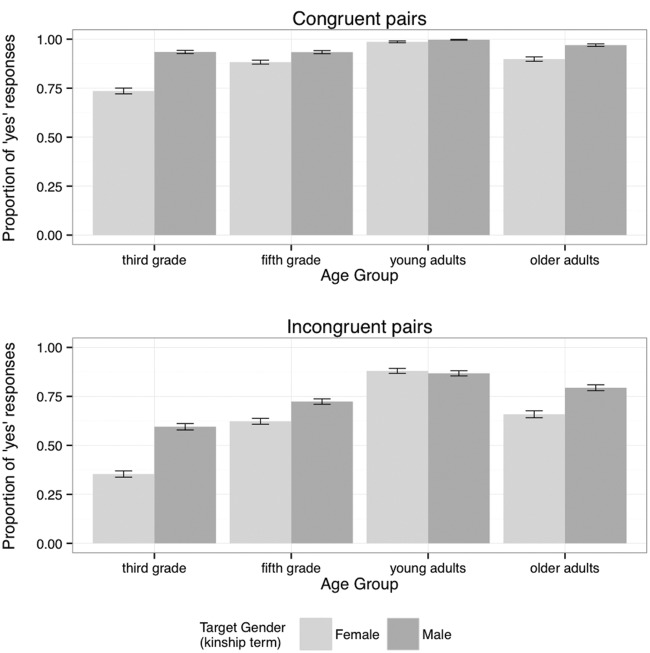
**Proportions of ‘yes’ responses for congruent and incongruent prime-target pairs, by Age Group and Target Gender (mean and standard error)**.

To test our predictions that there will be age-dependent sensitivity to the violation of gender stereotypical information, and that there will be asymmetries in the acceptability of incongruent items depending on the gender of the items, we ran a model including as predictors Congruence, Age Group, and Target Gender, as well as Block^2^. **Figure 1** presents a summary of the proportions of ‘yes’ responses by Congruence, Age Group, and Target Gender.

The statistical model confirmed simple effects of Block (participants increasingly respond with ‘yes’ across blocks: χ^2^ = 63.90, df: 1, *p* < 0.0001), Congruence (the proportion of ‘yes’ responses was higher for congruent pairs: χ^2^ = 1166.22, df: 1, *p* < 0.0001), Target Gender (more ‘yes’ responses after pairs with male targets, χ^2^ = 15.18, df: 1, *p* < 0.0001) and Age Group (χ^2^ = 69.60, df: 3, *p* < 0.0001). The overall effect of Age Group reflects a very high level of ‘yes’ responses for young adults, with lower levels for old adults, then fifth-graders and, finally, third-graders. As well as demonstrating an overall effect, Congruence was involved in a number of interactions. Therefore, we subsequently modeled congruent and incongruent conditions separately, with Block, Target Gender, and Age Group as predictors.

### Congruent Pairs

The analysis of congruent pairs revealed simple effects of Block (χ^2^ = 15.14, df: 1, *p* < 0.0001), Age Group (χ^2^ = 68.04, df: 3, *p* < 0.0001), and Target Gender (χ^2^ = 7.59, df: 1, *p* < 0.01), and a significant interaction of Target Gender and Age Group (χ^2^ = 25.36, df: 3, *p* < 0.0001). As can be seen from the upper panel of **Figure 1**, the interaction of Target Gender and Age Group reflects the fact that there were considerably more ‘yes’ responses to male targets than to female targets in congruent pairs for third graders, with smaller Target Gender differences in the same direction for older adults and fifth graders, and virtually no difference for young adults.

Subsequent analysis of each age group in the congruent condition showed no effects for young adults; this is hardly surprising given the ceiling-level performance that is visible in the top panel of **Figure 1**. For the other three groups we were able to introduce Participant Sex into the models (see Footnote 2). For each group there was a significant interaction of Target Gender and Participant Sex (older adults: χ^2^ = 17.28, df: 1, *p* < 0.0001; third-graders: χ^2^ = 16.31, df: 1, *p* < 0.0001; fifth-graders: χ^2^ = 4.18, df: 1, *p* < 0.05). In each case, there was a larger Target Gender difference for male participants than for female participants for both of these groups. In addition, males gave fewer ‘yes’ responses than females when the target was female, but more when it was male (see **Table 3**).

**Table 3:** Proportion of ‘yes’ responses by Age Group, Participant Sex and Target Gender, congruent pairs.

	Female participants		Male participants	
	Female targets	Male targets	Female targets	Male targets
Third grade	0.79	0.92	0.68	0.95
Fifth grade	0.89	0.91	0.88	0.95
Young adults	0.99	1.00	0.98	0.99
Older adults	0.91	0.95	0.89	0.99

### Incongruent Pairs

As with the congruent pairs, the analysis of the incongruent pairs revealed a significant interaction of Target Gender and Age Group (χ^2^ = 35.60, df: 3, *p* < 0.0001). In addition, there were significant simple effects of Block (χ^2^ = 44.13, df: 1, *p* < 0.0001), Age Group (χ^2^ = 49.79, df: 3, *p* < 0.0001), and Target Gender (χ^2^ = 4.46, df: 1, *p* < 0.05). The lower panel of **Figure 1** shows that the interaction of Target Gender and Age Group is similar to that found for the congruent pairs, but is more strongly marked. The largest difference for Target Gender is for the third graders, followed by older adults, then fifth graders and finally young adults, who have a very small difference in the opposite direction. As before, each of these age groups was subsequently analyzed in separate models, which included Participant Sex. The young adult data showed no effects of Participant Sex or Target Gender. The older adults showed a significant effect for Target Gender (χ^2^ = 4.34, df: 1, *p* < 0.05), and this effect was marginally significant for the fifth-graders (χ^2^ = 3.37, df: 1, *p* = 0.07). A major difference in the case of the third graders is that there was a significant interaction of Target Gender and Participant Sex (χ^2^ = 29.37, df: 1, *p* < 0.0001), as well as a significant simple effect for Target Gender (χ^2^ = 9.61, df: 1, *p* < 0.005). **Table 4** shows that, although both third-grade boys and girls gave more ‘yes’ responses to incongruent items that had a male kinship term as the target, this difference was over twice as large for boys as for girls.

**Table 4:** Proportion of ‘yes’ responses by Age Group, Participant Sex and Target Gender, incongruent pairs.

	Female participants		Male participants	
	Female targets	Male targets	Female targets	Male targets
Third grade	0.37	0.51	0.33	0.67
Fifth grade	0.61	0.68	0.63	0.76
Young adults	0.92	0.89	0.84	0.85
Older adults	0.65	0.77	0.67	0.82

### Response Choice Data: Summary

Overall, congruent pairs led to higher levels of ‘yes’ responses (responding that the prime and target could describe the same person) than incongruent pairs. This is true of all age groups, supporting our prediction that all groups will show sensitivity to stereotype violation. Importantly, though, this effect varies across age groups, and is most marked with the younger children, indicating that they have the lowest level of stereotype flexibility.

The incongruent pairs showed strong age-related effects, as well as Target Gender effects, with male targets following female role nouns receiving higher proportions of ‘yes’ responses than female targets, particularly from the younger children. This finding is contrary to our prediction that combinations of male roles with female kinship terms will be more acceptable than vice versa. Note though that a similar Target Gender difference was identified in the congruent condition. We also found interactions of Participant Sex and Target Gender for congruent pairs for all age groups except for the young adults, but only for the third-graders for the incongruent pairs. We will return to these findings in the Discussion section.

### Response Times

The dependent variable in this analysis was the set of transformed response times (using the inverse square root transformation). For clarity, however, the graphs below present the untransformed mean response times. Two sets of analyses were carried out, one for the ‘yes’ responses and one for the ‘no’ responses. The second of these included only responses to incongruent pairs, because the low numbers of ‘no’ responses to congruent pairs in some combinations of predictors made it difficult to obtain reliable regression models (see Footnote 2).

### ‘Yes’ Responses

Our initial analysis included the predictors Congruence, Age Group, Participant Sex, Target Gender, and Block. This revealed a significant three-way interaction of Age Group, Participant Sex, and Target Gender (χ^2^ = 22.25, df: 3, *p* < 0.0001), significant two-way interactions of Participant Sex and Target Gender (χ^2^ = 39.96, df: 1, *p* < 0.0001) and Age Group and Congruence (χ^2^ = 9.72, df: 3, *p* < 0.05), and simple effects of Block (χ^2^ = 19.87, df: 1, *p* < 0.0001), Congruence (χ^2^ = 255.63, df: 1, *p* < 0.0001), and Age Group (χ^2^ = 51.62, df: 3, *p* < 0.0001).

**FIGURE 2:**
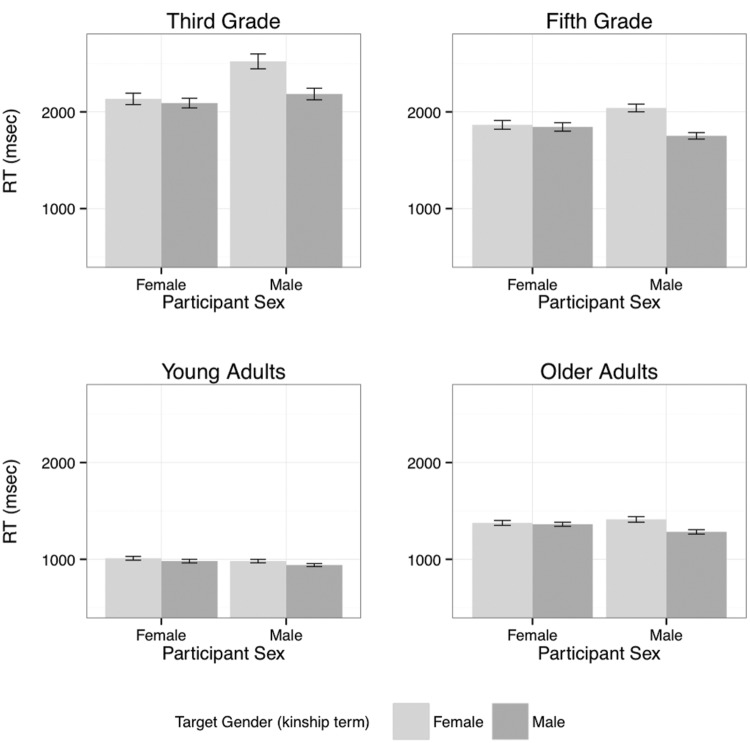
**Response times for ‘yes’ responses by Age Group, Participant Sex, and Target Gender (mean and standard error)**.

**FIGURE 3:**
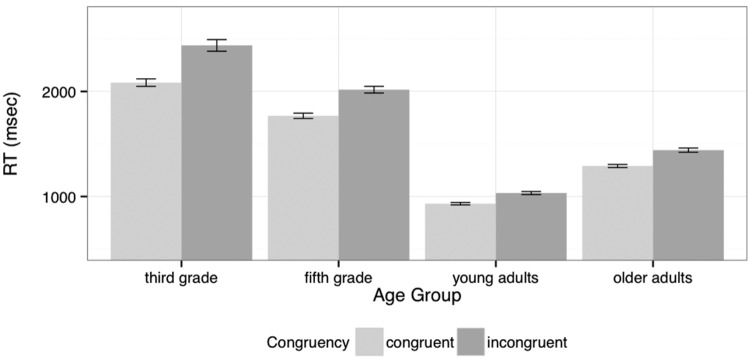
**Response times for ‘yes’ responses by Age Group and Congruence (mean and standard error)**.

**Figure 2** illustrates the three-way interaction between Age Group, Participant Sex, and Target Gender – the different age groups clearly show different effects of the interaction of Participant Sex and Target Gender. The overall effect of Age Group is also obvious in this figure. **Figure 3** presents the interaction of Age Group and Congruence and shows how the Congruence effect differs in size but not in direction across the groups. That is, all groups more readily accept stereotype-matching pairs than incongruent pairs, with this effect stronger for the children and smallest for the young adults. To explore the two interaction effects involving Age Group, separate analyses were carried out for each group.

For the young adults, the only significant effects were for Block (χ^2^ = 24.20, df: 1, *p* < 0.0001) and Congruence (χ^2^ = 27.18, df: 1, *p* < 0.0001). These young adults were faster in accepting congruent pairs than in accepting incongruent pairs, and their responses sped-up across the experiment. The older adult group similarly demonstrated significant effects for Block (χ^2^ = 30.56, df: 1, *p* < 0.0001) and Congruence (χ^2^ = 90.35, df: 1, *p* < 0.0001), but also a significant interaction of Participant Sex and Target Gender (χ^2^ = 10.90, df: 1, *p* < 0.001). This interaction arises because the male participants responded more quickly to male than to female targets, while the female participants showed no difference (see **Figure 2**).

The older of the two groups of children showed a significant interaction between Participant Sex and Target Gender (χ^2^ = 18.29, df: 1, *p* < 0.0001) and a simple effect of Congruence (χ^2^ = 114.44, df: 1, *p* < 0.0001), with no other effects. The interaction of Participant Sex and Target Gender has the same pattern as reported above for the older adults. The third grade participants also showed this significant interaction of Participant Sex and Target Gender (χ^2^ = 29.80, df: 1, *p* < 0.0001), as well as simple effects of Block (χ^2^ = 4.25, df: 1, *p* < 0.05) and Congruence (χ^2^ = 65.54, df: 1, *p* < 0.0001). The Block effect reflects a slowing-down as the experiment progressed (see Footnote 1). As with the older adults and fifth graders, the interaction of Participant Sex and Target Gender arises because male participants responded faster to male than to female targets, while females responded equally fast to both.

### ‘No’ Responses

As noted above, the analysis of ‘no’ responses included only the incongruent pairs. This analysis showed significant interactions of Target Gender with Age Group (χ^2^ = 8.53, df: 3, *p* < 0.05) and with Participant Sex (χ^2^ = 4.79, df: 3, *p* < 0.05), a significant interaction of Age Group with Block (χ^2^ = 22.47, df: 3, *p* < 0.0001), and an overall simple effect of Age Group (χ^2^ = 11.25, df: 3, *p* < 0.01).

**FIGURE 4:**
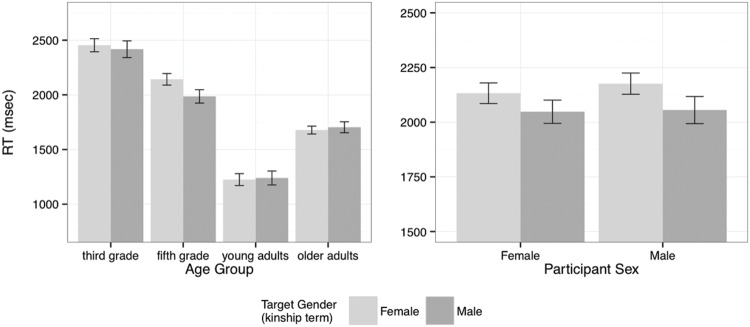
**Response times for ‘no’ responses to incongruent items (mean and standard error).** The left panel shows the interaction of Target Gender and Age Group and the right panel shows the interaction of Target Gender and Participant Sex.

The interactions of Target Gender with Age Group and Participant Sex are illustrated in the left and right panels of **Figure 4** respectively. In the left panel we see that the fifth grade children gave faster ‘no’ responses to incongruent pairs that involved a male target following a female prime, while the adult groups and the third grade children showed no such difference. This pattern was confirmed in further analyses for each age group: the young and older adults and the third graders showed no effect of Target Gender (all *p*s > 0.7), whereas the difference was significant for the fifth graders (χ^2^ = 4.17, df: 1, *p* < 0.05). The interaction of Target Gender with Participant Sex shown in the right panel is one of degree rather than of direction (contrast the interaction effects for these variables in the analysis of ‘yes’ response times) – both male and female participants gave faster ‘no’ responses to incongruent pairs involving a male target (following a female prime), but this difference was larger for the male participants.

### Response Time Data: Summary

Overall, young adults responded fastest, followed by older adults, fifth graders and lastly third graders. All groups were faster in accepting congruent than incongruent pairs. The groups differed from one another in the relative effects on ‘yes’ response times of the interaction of Participant Sex and Target Gender. This interaction arose because while there was little difference in the response times of female participants that depended on the gender of the kinship term used as the target noun, responses from male participants were faster to male targets than to female targets (reflecting the increased level of ‘yes’ responses after these targets noted above). This difference was strongest for the third graders, followed by the fifth graders and the older adults, but the effect was not significant for the young adults. The interaction of Participant Sex and Target Gender in the ‘no’ responses to incongruent pairs shows a similar pattern, that is, a stronger difference between responses to male and female targets from the male participants.

## Discussion

In a timed decision task, Italian third- and fifth-graders, young adults, and older adults were required to decide as quickly as possible if two auditorily presented words – a masculine or feminine stereotypical word combined with either a male or a female kinship term – could be used to describe the same person. Participants across all age groups were significantly more likely to respond ‘yes’ and to do so more rapidly when the kinship term was preceded by a stereotypically congruent than incongruent role noun. These results provide evidence that language users of various ages – school-age children, young and older adults – are biased by gender stereotypes when making judgments about the likely identity of people fulfilling certain roles. In addition, the higher processing cost of responding to incongruent pairings of roles and kinship terms is reflected in the response time differences – across all age groups – between incongruent and congruent conditions.

Our results are in line with those of Oakhill et al. (2005), as well as those of a range of studies using a variety of behavioral, eye-tracking and ERP techniques, predominantly with young adults (Carreiras et al., 1996; Garnham et al., 2002; Duffy and Keir, 2004; Cacciari and Padovani, 2007; Most et al., 2007; Kreiner et al., 2008; Pyykkönen et al., 2010; Siyanova-Chanturia et al., 2012). Importantly, our study extends the evidence-base for automatic gender stereotype effects to children and older adults, and highlights the contribution that on-line measures can make to the assessment of gender stereotyping across ages. In contrast to more traditional off-line measures, which have been widely used in studies with children and older adults, on-line measures are based largely on automatic processes that are believed to be free of strategic responses.

In addition to finding a general effect of gender stereotypes across ages, we also discovered a number of important differences in the processing of stereotypical gender (in)congruencies in children and adults. As noted in our introduction, Radvansky et al. (2010) suggested that older adults might not show the drop-off in stereotype flexibility (operationalised as an inability to suppress gender stereotypes) that has been reported for racial stereotypes. However, taking the proportion of ‘yes’ responses to incongruent pairs as one measure and the speed with which such ‘yes’ responses are made as another, we see that older adults, like the two groups of children, show lowered levels of stereotype flexibility, that is, of being able to identify that a stereotype can be wrong. In line with Devine (1989), von Hippel et al. (2000) and Gonsalkorale et al. (2009), we take our results to support the idea that older individuals are less likely to suppress their prejudiced behavior and are less able to regulate automatically activated associations when compared to younger adults. Our results thus appear to go against those reported in Radvansky et al. (2009) who found no reliable differences between young and older adults’ ability to discount gender stereotypical bias. It should be noted, however, that in Radvansky et al. (2009), counter-stereotypic information was *explicitly* provided to the participants, while in our study, no such information was present.

Within our two groups of children, we find results that are compatible with developmental stages of stereotype acquisition, in particular, with an increase in stereotype flexibility between the ages of 5 and 11 (Signorella et al., 1993; Trautner et al., 2005; Banse et al., 2010). Our 8-year-olds were found to be less flexible, less likely to press ‘yes’ following a stereotypically incongruent word pair, and slower in doing so than our 10-year-olds. The latter made ‘yes’ choices at a level comparable to that of the older adults, although the children’s responses were slower (as were their ‘yes’ responses to congruent pairs).

Another interesting set of effects pertains to the gender of the target (kinship) word. This is the asymmetry in the processing of incongruent pairs, predominantly in the data from our children and older adults, which favored the pairing of female roles with male kinship terms. This asymmetry is in the opposite direction to that predicted on the basis of previous results with children (Wilbourn and Kee, 2010) and young adults (Cacciari and Padovani, 2007; Siyanova-Chanturia et al., 2012; Reali et al., 2014). Note, however, that we further found that male targets in the congruent condition also received more and faster ‘yes’ responses than female targets for these groups. In other words, this asymmetry affects more than just the processing of incongruent pairs. In addition, we found participant sex differences in the decision choice and response times of the same three groups across both congruent and incongruent conditions, in interaction with these target gender effects. First, we found larger differences between the proportions of ‘yes’ responses to male and female targets for the male children and older adults than for their female counterparts, with the males providing fewer ‘yes’ responses than the females after female kinship terms, but more ‘yes’ responses than the females after male kinship terms. Second, these groups differed in how quickly female and male participants pressed ‘yes’ following female and male kinship terms. While female participants’ response times did not differ with the gender of the kinship term, male participants’ responses to male targets were faster than to female targets. In line with these findings, the analysis of the ‘no’ responses to incongruent pairs further suggested a bigger difference in responses to male and female targets for male than female participants.

How can we explain such gender asymmetries? We interpret the fact that male children and older adults responded more quickly to male kinship items, and their tendency, when compared to female participants, to prefer male kinship terms, as a reflection of the use of the social category “male” as the standard – or unmarked normative group – against which other categories are judged. According to social psychologists, one group (males) can become more “normative” than another (females), being the unmarked normative group (Hegarty and Pratto, 2001). For example, Miller et al. (1991) showed that when asked to think of a prototypical voter, most people think of a male voter exemplar. Researchers have argued that such “androcentrism” is common (Bem, 1993; Hegarty and Pratto, 2001), and that attitudes, beliefs, and stereotypes are more influenced by male exemplars than female ones (Eagly and Kite, 1987). It seems that social “androcentrism” affects male and female children and older adults differently, in that females, being members of the marked normative group, may be more sensitive and able to correct for the bias than males, being members of the unmarked normative group. Interestingly, no such effect was observed for our young adults, implying that age plays an important role in one’s ability to correct for the “unmarked group effect” and to inhibit stereotypical representations.

In addition, the gender asymmetry reflected in interactions of Target Gender and Participant Sex appears to be consistent with the claim of Miller et al. (2009) that boys generally have stronger stereotypical biases than girls, especially in the domain of activities. According to Miller et al. (2009), girls tend to confirm less strictly than boys to gender-role stereotypes. Interestingly, Miller et al. (2009) also maintain that gender stereotypes are differentially accessible when children think about males and females. These authors, as well as others (Higgins and King, 1981; Higgins, 1996), define accessibility as the readiness with which a construct is retrieved from memory. Our findings suggest that female children are equally fast to access and accept male and female constructs (kinship terms), while males more rapidly access male constructs than female ones.

In summary, our findings support the view according to which information about the stereotypical gender associated with occupations is incorporated into the representation of words denoting these occupations and is activated as soon as such a word is encountered. Importantly, the present study has gone beyond young adults to unveil the mechanisms of on-line processing of gender stereotypical information, as well as notable gender asymmetries associated with such processing, in two under-researched age groups – school-age children and cognitively preserved older adults.

## Conflict of Interest Statement

The authors declare that the research was conducted in the absence of any commercial or financial relationships that could be construed as a potential conflict of interest.

## Footnotes

1 Block was included because of widely-reported speeding-up and slowing-down effects across response time experiments which can add variance to response data. The block effects are not important in the context of our predictions above and will not be discussed in detail. However, it is interesting to note that in the response time analyses adults typically sped up across the experiment but the children slowed down. Importantly, additional analyses showed that these age-related block effects did not interact with the experimental variables in this study, suggesting that our participant groups did not change their performance strategically in response to patterns they may have noticed in the stimuli.

2 This initial analysis did not also include Participant Sex, because models including this as a predictor failed to converge. This is because the female young adults produced 100% ‘yes’ responses to subsets of data, a situation which produces inflated standard error in logistic regression. Since our prediction concerning Participant Sex relates primarily to children rather than adults, this factor will be reintroduced in subsequent analyses of each age group.

## References

[B1] Banaji M. Hardin C. D. (1996). Automatic stereotyping. *Psychol. Sci.* 7 136–141. 10.1111/j.1467-9280.1996.tb00346.x

[B2] Banse R. Gawronski B. Rebetez C. Gutt H. Morton B. (2010). The development of spontaneous gender stereotyping in childhood: relations to stereotype knowledge and stereotype flexibility. *Dev. Sci.* 13 298–306. 10.1111/j.1467-7687.2009.00880.x 20136926

[B3] Baroni M. Bernardini S. Comastri F. Piccioni L. Volpi A. Aston G. (2004). “Introducing the “la Repubblica” corpus: a large, annotated, TEI(XML)-compliant corpus of newspaper Italian,” in *Proceedings of LREC* 2004 Lisbon: European Language Resources Association.

[B4] Bates D. Maechler M. Bolker B. Walker S. (2015). *lme4: Linear Mixed-Effects Models using Eigen and S4. R Package Version 1.1-8.* Available at: http://CRAN.R-project.org/package=lme4

[B5] BBC News (2013). Available at: http://www.bbc.co.uk/news/uk-19657646 [accessed March 24, 2013].

[B6] Bem S. L. (1993). *The Lenses of Gender.* New Haven, CT: Yale University Press.

[B7] Bornkessel-Schlesewsky I. Schlesewsky M. (2008). An alternative perspective on “semantic P600” effects in language comprehension. *Brain Res. Rev.* 59 55–73. 10.1016/j.brainresrev.2008.05.003 18617270

[B8] Cacciari C. Padovani R. (2007). Further evidence of gender stereotype priming in language: semantic facilitation and inhibition in Italian role nouns. *Appl. Psycholinguist.* 28 277–293. 10.1017/S0142716407070142

[B9] Carreiras M. Garnham A. Oakhill J. Cain K. (1996). The use of stereotypical information in constructing a mental model: evidence from English and Spanish. *Q. J. Exp. Psychol.* 49A 639–663. 10.1080/713755647 8828401

[B10] Devine P. G. (1989). Stereotypes and prejudice: their automatic and controlled components. *J. Pers. Soc. Psychol.* 56 5–18. 10.1037/0022-3514.56.1.5

[B11] Diekman A. B. Eagly A. H. (2000). Stereotypes as dynamic constructs: women and men of the past, present, and future. *Pers. Soc. Psychol. Bull.* 26 1171–1188. 10.1177/0146167200262001

[B12] Duffy S. A. Keir J. A. (2004). Violating stereotypes: eye movements and comprehension processes when text conflicts with world knowledge. *Mem. Cognit.* 32 551–559. 10.3758/BF03195846 15478749

[B13] Eagly A. Kite M. (1987). Are stereotypes of nationalities applied to both men and women? *J. Pers. Soc. Psychol.* 53 457–462. 10.1037/0022-3514.53.3.451

[B14] Eagly A. H. Steffen V. J. (1984). Gender stereotypes stem from the distribution of women and men into social roles. *J. Pers. Soc. Psychol.* 46 735–754. 10.1037/0022-3514.46.4.735

[B15] Eagly A. H. Wood W. Diekman A. B. (2000). “Social role theory of gender differences and similarities: a current appraisal,” in *The Developmental Social Psychology of Gender* eds Eckes T. Trautner H. M. (Mahwah: Lawrence Erlbaum) 123–174.

[B16] Esaulova Y. Reali C. von Stockhausen L. (2014). Influences of grammatical and stereotypical gender during reading: eye movements in pronominal and noun phrase anaphor resolution. *Lang. Cogn. Neurosci.* 29 781–803. 10.1080/01690965.2013.794295

[B17] Folstein M. F. Folstein S. E. McHugh P. R. (1975). “Mini-mental state”: a practical method for grading the cognitive state of patients for the clinician. *J. Psychiatr. Res.* 12 189–198. 10.1016/0022-3956(75)90026-6 1202204

[B18] Garnham A. Oakhill J. Reynolds D. (2002). Are inferences from stereotyped role names to characters’ gender made elaboratively? *Mem. Cognit.* 30 439–446. 10.3758/BF03194944 12061764

[B19] Gonsalkorale K. Sherman J. Klauer K. C. (2009). Aging and prejudice: diminished regulation of automatic race bias among older adults. *J. Exp. Soc. Psychol.* 45 410–414. 10.1016/j.jesp.2008.11.004

[B20] Hegarty P. Pratto F. (2001). The effects of social category norms and stereotypes on explanations for intergroup differences. *J. Pers. Soc. Psychol.* 80 723–735. 10.1037/0022-3514.80.5.723 11374745

[B21] Higgins E. T. (1996). “Knowledge activation: accessibility, applicability, and salience,” in *Social Psychology: Handbook of Basic Principles* eds Higgins E. T. Kruglanski A. W. (New York, NY: Guilford) 133–168.

[B22] Higgins E. T. King G. (1981). “Accessibility of social constructs: information processing consequences of individual and contextual variability,” in *Personality, Cognition and Social Interaction* eds Cantor N. Kihlstrom J. F. (Hillsdale, MI: Erlbaum) 69–121.

[B23] Hill S. Flom R. (2007). 18- and 24-month-olds’ discrimination of gender-consistent and inconsistent activities. *Infant Behav. Dev.* 30 168–173. 10.1016/j.infbeh.2006.08.003 17292790

[B24] Irmen L. (2007). What’s in a (role) name? Formal and semantic aspects of comprehending personal nouns. *J. Psycholinguist. Res.* 36 431–456. 17372839 10.1007/s10936-007-9053-z

[B25] Irmen L. Holt D. V. Weisbrod M. (2010). Effects of role typicality on processing person information in German: evidence from an ERP study. *Brain Res.* 1353 133–144. 10.1016/j.brainres.2010.07.018 20637743

[B26] Kim A. Osterhout L. (2005). The independence of combinatory semantic processing: evidence from event-related potentials. *J. Mem. Lang.* 52 205–225. 10.1016/j.jml.2004.10.002

[B27] Kreiner H. Sturt P. Garrod S. (2008). Processing definitional and stereotypical gender in reference resolution: evidence from eye-movements. *J. Mem. Lang.* 58 239–261. 10.1016/j.jml.2007.09.003

[B28] Kuperberg G. R. Sitnikova T. Caplan D. Holcomb P. J. (2003). Electrophysiological distinctions in processing conceptual relationships within simple sentences. *Cogn. Brain Res.* 17 117–129. 10.1016/S0926-6410(03)00086-7 12763198

[B29] Kutas M. Federmeier K. D. (2011). Thirty years and counting: finding meaning in the N400 component of the event related brain potential (ERP). *Annu. Rev. Psychol.* 62 621–647. 10.1146/annurev.psych.093008.131123 20809790 PMC4052444

[B30] Miller C. Lurye L. Zosuls K. Ruble D. (2009). Accessibility of gender stereotypes domains: developmental and gender differences in children. *Sex Roles* 60 870–881. 10.1007/s11199-009-9584-x 19606278 PMC2709873

[B31] Miller D. T. Taylor B. Buck M. L. (1991). Gender gaps: who needs to be explained? *J. Pers. Soc. Psychol.* 61 5–12. 10.1037/0022-3514.61.1.5 1890588

[B32] Most S. Sorber A. V. Cunningham J. (2007). Auditory Stroop reveals implicit gender associations in adults and children. *J. Exp. Soc. Psychol.* 43 287–294. 10.1016/j.jesp.2006.02.002

[B33] Oakhill J. V. Garnham A. Reynolds D. J. (2005). Immediate activation of stereotypical gender information. *Mem. Cognit.* 33 972–983. 10.3758/BF03193206 16496719

[B34] Osterhout L. Bersick M. McLaughlin J. (1997). Brain potentials reflect violations of gender stereotypes. *Mem. Cognit.* 25 273–285. 10.3758/BF03211283 9184479

[B35] Osterhout L. Holcomb P. J. (1992). Event-related brain potentials elicited by syntactic anomaly. *J. Mem. Lang.* 31 785–806. 10.1016/0749-596X(92)90039-Z

[B36] Osterhout L. Holcomb P. J. (1995). “Event-related brain potentials and language comprehension,” in *Electrophysiology of Mind: Event-Related Brain Potentials and Cognition* eds Rugg M. D. Coles M. G. H. (Oxford: Oxford University Press).

[B37] Poulin-Dubois D. Serbin L. Eichstedt J. Sen M. (2002). Men don’t put on make-up: toddlers’ knowledge of the gender stereotyping of household activities. *Soc. Dev.* 11 166–181. 10.1111/1467-9507.00193

[B38] Pyykkönen P. Hyönä J. Van Gompel R. P. G. (2010). Activating gender stereotypes during online spoken language processing: evidence from visual world eye tracking. *Exp. Psychol.* 57 126–133. 10.1027/1618-3169/a000016 20178931

[B39] Radvansky G. Copeland D. von Hippel W. (2010). Stereotype activation, inhibition, and aging. *J. Exp. Soc. Psychol.* 46 51–60. 10.1016/j.jesp.2009.09.010 20161549 PMC2805127

[B40] Radvansky G. Lynchard N. von Hippel W. (2009). Aging and stereotype suppression. *Aging Neuropsychol. Cogn.* 16 22–32. 10.1080/13825580802187200 18608050

[B41] Reali C. Esaulova Y. von Stockhausen L. (2014). Isolating stereotypical gender in a grammatical gender language: evidence from eye movements. *Appl. Psycholinguist.* 36 977–1006. 10.1017/S0142716414000010

[B42] Signorella M. L. Bigler R. S. Liben L. S. (1993). Developmental differences in children’s gender schemata about others: a meta-analytic review. *Dev. Rev.* 12 147–183. 10.1006/drev.1993.1007

[B43] Singmann H. Bolker B. Westfall J. Højsgaard S. Fox J. Lawrence M. A. (2015). *afex: Analysis of Factorial Experiments, vsn. 0.13-145.* Available at: https://cran.r-project.org/web/packages/afex/index.html

[B44] Siyanova-Chanturia A. Pesciarelli F. Cacciari C. (2012). The electrophysiological underpinnings of processing gender stereotypes in language. *PLoS ONE* 7:e48712 10.1371/journal.pone.0048712 PMC3513306 23226494

[B45] Trautner N. M. Ruble D. N. Cyphers L. Kirsten B. Behrendt R. Hartmann P. (2005). Rigidity and flexibility of gender stereotypes in childhood: developmental or differential? *Infant Child Dev.* 14 365–381. 10.1002/icd.399

[B46] Van Berkum J. J. A. Van den Brink D. Tesink C. M. J. Y. Kos M. Hagoort P. (2008). The neural integration of speaker and message. *J. Cogn. Neurosci.* 20 580–591. 10.1162/jocn.2008.20054 18052777

[B47] von Hippel W. Silver L. Lynch M. (2000). Stereotyping against your will: the role of inhibitory ability in stereotyping and prejudice among the elderly. *Pers. Soc. Psychol. Bull.* 26 523–532. 10.1177/014616720026700

[B48] White K. R. Crites S. L. Taylor J. H. Corral G. (2009). Wait, what? Assessing stereotype incongruities using the N400 ERP component. *Soc. Cogn. Affect. Neurosci.* 4 191–198. 10.1093/scan/nsp004 19270040 PMC2686231

[B49] Wilbourn M. P. Kee D. W. (2010). Henry the nurse is a doctor too: implicitly examining children’s gender stereotype flexibility for male and female occupational roles. *Sex Roles* 62 670–683. 10.1007/s11199-010-9773-7

