# Supplementary material for: Gender stereotypes across the ages: On-line processing in school-age children, young and older adults
Source: Front Psychol. 2015 Sep 22;6:1388. doi: 10.3389/fpsyg.2015.01388 (PMC4585124; doi:10.3389/fpsyg.2015.01388)
Supplement: Supplementary file 1 [file Data_Sheet_1.DOCX]

**Appendix**

*Italian male-oriented and female-oriented stereotypical role nouns with English translations.*

| **Male-oriented stereotype** | | **Female-oriented stereotype** | |
| --- | --- | --- | --- |
| **Italian** | **English** | **Italian** | **English** |
| *batterista* | “drummer” | *babysitter* | “babysitter” |
| *camionista* | “truck driver” | *badante* | “social care worker “ |
| *mercante* | “merchant” | *farmacista* | “pharmacist” |
| *ciclista* | “cyclist” | *fiorista* | “florist” |
| *paracadutista* | “parachutist” | *insegnante* | “school teacher” |
| *preside* | “headmaster” | *pallavolista* | “volleyball player” |
| *autista* | “driver” | *stilista* | “stylist” |
| *chef* | “chef” | *supplente* | “substitute teacher” |
| *commerciante* | “trader” | *violinista* | “violinist” |
